# The risk factors of gestational hypertension in patients with polycystic ovary syndrome: a retrospective analysis

**DOI:** 10.1186/s12884-021-03808-3

**Published:** 2021-04-27

**Authors:** Shu Zhou, Yiping Ji, Haimei Wang

**Affiliations:** 1 Department of gynaecology, The 5th Affiliated Hospital of Nantong University (Taizhou People’s Hospital), Taizhou, China; 2 Department of gynaecology, Lian Shui county People’s Hospital, Huai’an, China; 3 Department of Urology, The Affiliated Huai’an Hospital of Xuzhou Medical University and The Second People’s Hospital of Huai’an, No.62, Huaihai Road, Jiangsu 223002 Huai’an, China

**Keywords:** Hypertension, Pregnancy, Polycystic ovary syndrome, Prevention, Care

## Abstract

**Background:**

The hypertensive disorders complicating pregnancy (HDCP) is common in patients with polycystic ovary syndrome (PCOS), yet the potential influencing factors remained unclear. We aimed to assess the independent risk factors of HDCP in patients with PCOS, to provide clinical evidences for the management of PCOS.

**Methods:**

Pregnant PCOS patients treated in our hospital from June 1, 2018 to November 30, 2020 were approached. The personal and clinical characteristics of patients with and without gestational hypertension were evaluated. Logistic regressions were conducted to identify the independent risk factors of HDCP, Receiver operating characteristics (ROC)curve analysis was conducted to evaluate the predicting value.

**Results:**

A total of 188 PCOS patients were included, the incidence of HDCP in patients with PCOS was 27.66 %. There were significant differences in the age, BMI, family history of hypertension, the history of adverse pregnancy, history of contraceptive pills use and family history of HDCP between HDCP group and no-HDCP group (all *p* < 0.05), and there were no significant differences in the family history of diabetes, multiple pregnancy and long-term smoking history between HDCP group and no-HDCP group (all *p* > 0.05). Age ≥ 27y(OR2.048, 95 %CI1.121 ~ 3.208), BMI ≥ 24 kg/m^2^(OR1.463, 95 %CI1.069 ~ 2.011), family history of hypertension(OR2.129, 95 %CI1.093 ~ 3.042), the history of adverse pregnancy(OR2.435, 95 %CI1.264 ~ 4.085), history of contraceptive pills use(OR3.806, 95 %CI1.184 ~ 6.102), family history of HDCP(OR1.934, 95 %CI1.016 ~ 2.774) were the independent risk factors of HDCP in patients with PCOS (all *p* < 0.05). ROC curve analyses indicated that those factors had good predictive value on HDCP in PCOS patients.

**Conclusions:**

The incidence of HDCP in PCOS patients is relatively high. In clinical practice, medical workers should carry out early prevention and intervention measures for these risk factors to reduce the incidence of HDCP.

## Background

Polycystic ovary syndrome (PCOS) is one of the most common endocrine disorders in women of childbearing age, and it is also the main cause of anovulatory infertility [1]. PCOS is mainly manifested by menstrual disorders, hirsutism, infertility, and hyperandrogenism [2]. The prevalence of PCOS ranges from 5.11 to 10.08 %, which is related to different races, populations, regions, and diagnostic criteria [3, 4]. Previous studies [5, 6] have found that PCOS patients are prone to insulin resistance, hyperinsulinemia, cardiovascular disease, metabolic syndrome, sleep apnea, and hypertension during pregnancy. Currently, the pathogenesis of PCOS has not yet been fully elucidated [7]. Therefore, the prevention and treatment of PCOS is on the top research agenda of medical staff.

Hypertensive disorders complicating pregnancy (HDCP) especially the gestational hypertension is one of the common complications that are unique to pregnancy, and its specific pathogenesis is currently unclear, which can lead to a variety of adverse pregnancy outcomes and seriously harm the health of mothers and children [8]. It often occurs in the middle and late stages of pregnancy [9]. The blood pressure of patients during pregnancy continues to be higher than the upper limit of normal, which causes greater damage to multiple organs of the pregnant woman, but the specific cause is still unclear [10]. Many studies [11–13] believe that age, pre-pregnancy BMI, changes in blood viscosity, reduction in platelet count and other related risks have a significant role in promoting the occurrence of HDCP. Pregnancy is a dynamic development process, and the basic physical condition of pregnant women in early pregnancy and the influence of related factors during pregnancy can all lead to the occurrence of HDCP [14]. To this end, this study aimed to analyze the independent risk factors of HDCP in patients with PCOS, to provide evidence support for the prevention of HDCP in patients with PCOS, to improve the prognosis of PCOS patients.

## Methods

 Our study was a retrospective study design, and it had been checked and approved by the ethical committee of The Affiliated Huai’an Hospital of Xuzhou Medical University (No.18,020,277), and all the included patients had been well-informed and agreed to participant in this study, written informed consents had been obtained from all the patients. We tried to conduct and report this study according to related guidelines.

### Patients

We retrospectively selected the pregnant PCOS patients who were diagnosed and treated in our hospital from June 1, 2018 to November 30, 2020 as the research populations, the patients were selected from the antenatal department of our hospital. The inclusion criteria for this study were: those pregnant patients who underwent gestational hypertension screening in our hospital ; the diagnosis of PCOS met the relevant diagnostic criteria [15, 16]: ①rare ovulation or anovulation; ②clinical manifestations of hyperandrogen and (or) hyperandrogenemia; ③ovarian polycystic changes. PCOS was diagnosed when two of the previous three items are met, and the PCOS were confirmed through the lab pathology results and ultrasound. The patients were well-informed and agreed to participate in this study. The exclusion criteria of this study were: PCOS patients with previous tumors, rheumatic immune diseases, blood system and other diseases, and those with incomplete clinical data; the patients with chronic hypertension, fertility treatment; patients who do not agree to participate in this study.

### HDCP diagnosis

According to the HDCP diagnosis and treatment guideline [17] formulated by the HDCP Group of the Chinese Medical Association Obstetrics and Gynecology Branch, the gestational hypertension was diagnosed as follows: hypertension occur for the first time after 20 weeks of pregnancy, systolic blood pressure ≥ 140 mmHg and/or diastolic blood pressure ≥ 90 mmHg, returned to normal within 12 weeks after delivery; meanwhile, urine protein test was negative.

### Data collection

We reviewed and collected relevant medical data of patients undergoing prenatal check-ups in our hospital. The prenatal examinations in our hospital mainly include B-ultrasound to determine whether it is a normal intrauterine pregnancy and determine the gestational age; screening of unhealthy lifestyles, such as whether pregnant women smoke, drink, illegally use drugs, inject drugs, etc.; blood and urine routines and liver and kidney function analysis. cervical cytology; screening for Down syndrome. Two authors independently collected the personal and clinical characteristics of included PCOS patients through the medical records, including age, the booking body weight for body mass index (BMI), family history of hypertension, family history of diabetes, the history of adverse pregnancy, multiple pregnancy, history of contraceptive pills use, long-term smoking history, family history of HDCP.

### Statistical processing

We used SPSS 23.0 software for statistical analysis of relevant data. The measurement data was expressed by t test and expressed as mean ± standard deviation (x ± s); the count data was expressed by χ2 test and expressed by frequency(%). KS test was conducted to test the normality of analyzed outcomes, conditional mean completer method was performed to analyze the missing data. The variates with positive results in the univariate analysis were further included for logistic regression to identify the independent risk factors of HDCP, Receiver operating characteristics (ROC)curve analysis was conducted by PRISE Software package, and Youden index was calculated by the software. *P* < 0.05 indicated that the difference was statistically significant.

## Results

### Included patients

215 women’s medical record were initially screened, 27 patients were excluded because some of the personal characteristics were missing. Finally, a total of 188 PCOS patients were included, of whom 52 patients had been diagnosed as HDCP, the incidence of HDCP in patients with PCOS was 27.66 %. As presented in Table 1, there were significant differences in the age, BMI, family history of hypertension, the history of adverse pregnancy, history of contraceptive pills use and family history of HDCP between HDCP group and no-HDCP group (all *p* < 0.05), and there were no significant differences in the family history of diabetes, multiple pregnancy and long-term smoking history between HDCP group and no-HDCP group (all *p* > 0.05).

**Table 1:** The characteristics of included PCOS patients

Variables	HDCP group(*n* = 52)	No-HDCP group(*n* = 136)	t/χ^2^	p
Age(y)	28.81 ± 8.11	25. 05 ± 8.46	1.761	0.027
BMI(kg/m^2^)	24.97 ± 2.03	22.94 ± 1.89	1.024	0.021
Family history of hypertension	37(71.15 %)	25(18.38 %)	1.291	0.035
Family history of diabetes	10(19.23 %)	27(19.85 %)	1.744	0.092
The history of adverse pregnancy	28(53.85 %)	14(10.29 %)	1.607	0.017
Multiple pregnancy	9(17.30 %)	26(19.12 %)	1.812	0.069
History of contraceptive pills use	25(48.08 %)	25(18.38 %)	1.097	0.016
Long-term smoking history	6(11.54 %)	16(11.76 %)	1.182	0.083
Family history of HDCP	30(57.69 %)	22(16.18 %)	1.201	0.006

### The risk factors of HDCP in patients with PCOS

Table 2 presented the variable assignment of multivariate logistic regression. And as indicated in Table 3, Age ≥ 27y(OR2.048, 95 %CI1.121 ~ 3.208), BMI ≥ 24 kg/m^2^(OR1.463, 95 %CI1.069 ~ 2.011), family history of hypertension(OR2.129, 95 %CI1.093 ~ 3.042), the history of adverse pregnancy(OR2.435, 95 %CI1.264 ~ 4.085), history of contraceptive pills use(OR3.806, 95 %CI1.184 ~ 6.102), family history of HDCP(OR1.934, 95 %CI1.016 ~ 2.774) were the independent risk factors of HDCP in patients with PCOS (all *p* < 0.05).

**Table 2:** The variable assignment of multivariate logistic regression

Factors	Variables	Assignment
HDCP	Y	yes = 1, no = 2
Age(y)	X_1_	≥ 27 = 1, < 27 = 2
BMI(kg/m^2^)	X_2_	≥ 24 = 1, < 24 = 2
Family history of hypertension	X_3_	yes = 1, no = 2
The history of adverse pregnancy	X_4_	yes = 1, no = 2
History of contraceptive pills use	X_5_	yes = 1, no = 2
Family history of HDCP	X6	yes = 1, no = 2

**Table 3:** Logistic regression analysis on the risk factors of HDCP in patients with PCOS

Variables	Β	Wald	OR	95 %CI	p
Age ≥ 27y	0.158	12.129	2.048	1.121 ~ 3.208	0.042
BMI ≥ 24	0.122	7.199	1.463	1.069 ~ 2.011	0.025
Family history of hypertension	0.101	18.115	2.129	1.093 ~ 3.042	0.025
The history of adverse pregnancy	0.186	3.099	2.435	1.264 ~ 4.085	0.017
History of contraceptive pills use	0.123	17.173	3.806	1.184 ~ 6.102	0.023
Family history of HDCP	0.113	9.102	1.934	1.016 ~ 2.774	0.041

### The ROC analysis

**Fig. 1:**
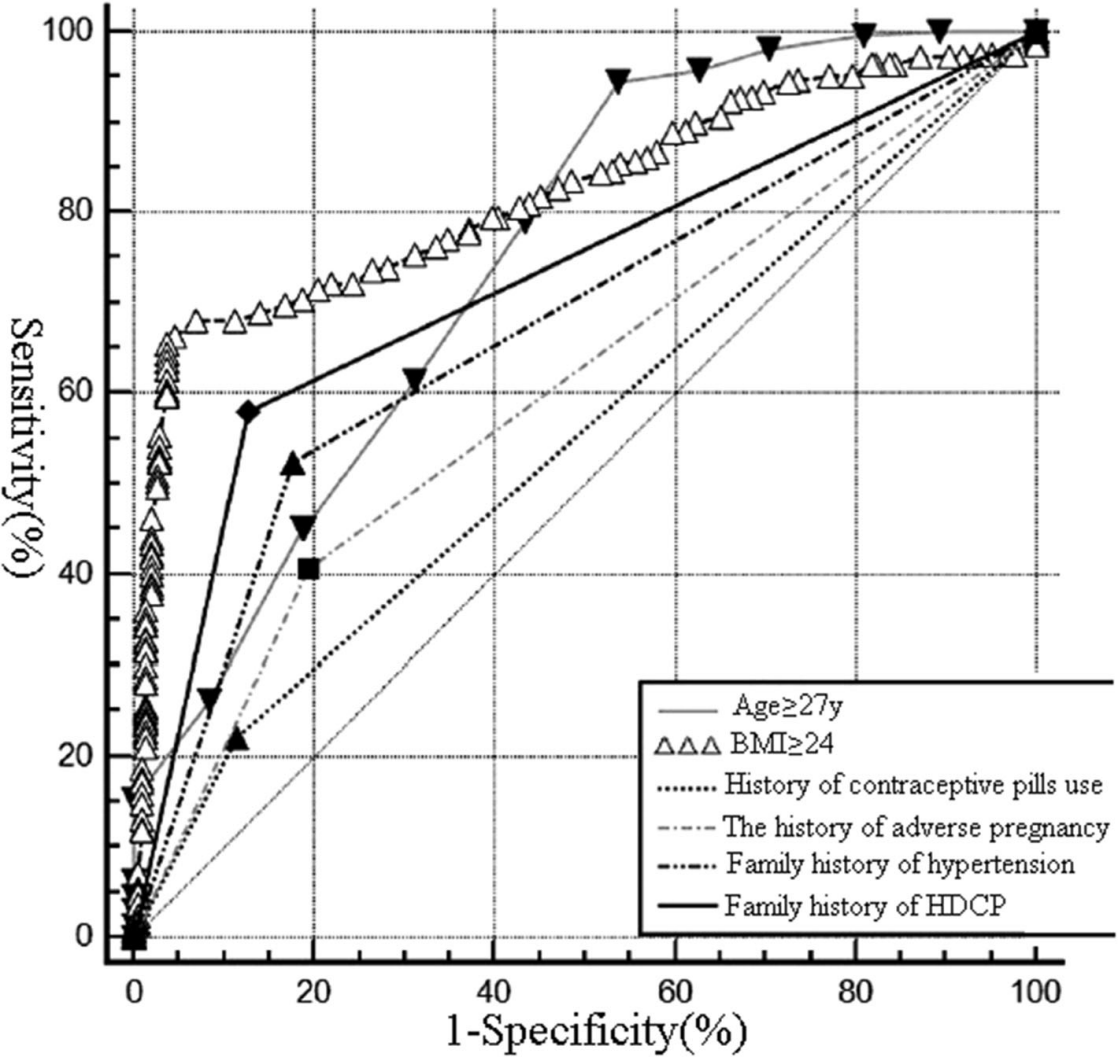
The ROC curve on the risk factors predicting the HDCP in PCOS patients

The ROC curve on the risk factors predicting the HDCP in PCOS patients was presented in Fig. 1. As presented in Table 4, age ≥ 27y, BMI ≥ 24, family history of hypertension, the history of adverse pregnancy, history of contraceptive pills use and family history of HDCP had good predictive value with good sensitivity and specificity for predicting the HDCP in PCOS patients.

**Table 4:** The reference value of related risk factors for HDCP in patients with PCOS

Variables	AUC	Yorden Index	Sensitivity	Specificity
Age ≥ 27y	0.725	0.388	94.65 %	46.36 %
BMI ≥ 24	0.828	0.602	65.55 %	96.13 %
Family history of hypertension	0.681	0.375	52.63 %	83.24 %
The history of adverse pregnancy	0.607	0.227	40.18 %	81.51 %
History of contraceptive pills use	0.548	0.186	69.19 %	81.57 %
Family history of HDCP	0.701	0.447	58.02 %	86.98 %

## Discussions

PCOS has a high incidence in countries around the world. Epidemiological studies [18–20]have reported that 30 % of women of childbearing age with abnormal BMI have the occurrence of PCOS. At the same time, the disease is also accompanied by a variety of metabolic-related diseases, which affect the whole body. Moreover, it has a certain promoting effect on other diseases such as endometrial cancer and hypertension during pregnancy [21]. The specific mechanism of PCOS is still not very clear. Some studies [8, 22, 23] believe that the occurrence of the disease is related to environmental factors. Various factors act to activate related signaling pathways, which in turn leads to abnormalities in various metabolic pathways, but metabolic abnormalities are related to the occurrence of PCOS. However, the connection between them is still unclear. Therefore, it is necessary to conduct further investigations on the mechanism and treatment strategies of PCOS.

HDCP is a special disease that occurs during pregnancy, with an incidence rate of about 9 % in China, which is higher than that of being reported in other countries [5, 24]. HDCP is one of the five major causes of maternal death during pregnancy. It often manifests as transient hypertension, proteinuria, etc., and the above symptoms and signs will disappear after delivery [25]. At present, related studies [26–28] believe that the possible causes of the disease are immune system imbalance, shallow placental implantation, abnormal trophoblast cells invading the myometrium, damage to the endovascular screen cells, and genetic factors. The incidence of HDCP in women with PCOS is much higher than that of ordinary healthy women of childbearing age, which is related to endocrine disorders, obesity, insulin resistance, etc. in patients with PCOS [29, 30], but there are few relevant research reports and lack of related risks analysis. The results of our study have found that the incidence of HDCP in patients with PCOS was 27.66 %, and age ≥ 27y, BMI ≥ 24, family history of hypertension, the history of adverse pregnancy, history of contraceptive pills use and family history of HDCP were the risk factors of HDCP in PCOS patients, PCOS patients with those risk factors should be highly alerted for HDCP.

The ability of independent risk factors to predict HDCP is different. The highest AUC and highest specificity are seen in the BMI. Previous related studies[31, 32] have shown that abortion history and age are independent risk factors that affect the pregnancy outcome of patients with polycystic ovary syndrome, which is consistent with the conclusions of this study. Previous studies[33, 34] also have confirmed that BMI, age, family history of hypertension, etc. are independent risk factors that affect the onset of hypertension during pregnancy, which is also consistent with the findings of this study.

Many recent studies[35, 36] have found that insulin resistance is one of the pathogenesis of HDCP. Vascular artery smooth muscle cells and vascular endothelial cells are both insulin-sensitive cells. Vascular endothelial cells are activated under the stimulation of hyperinsulinemia and cause further damage. Nitric oxide production is reduced, while prostaglandin production is inhibited, and peripheral vascular resistance increase, eventually leading to increased blood pressure[37, 38]. In addition, hyperinsulinemia can also promote abnormal vascular smooth muscle cell proliferation, vascular endothelial dysfunction, vascular lumen stenosis, and increased vascular resistance[39]. Since most PCOS patients show lipid peroxidation and hyperlipidemia, this may also be one of the reasons for the damage of vascular endothelial cell function[40]. Pathophysiological studies have shown that insulin resistance plays a role in promoting the early stages of hypertension in pregnancy, such as vascular endothelial damage and hyperlipidemia, which may eventually lead to the development of hypertension in pregnancy[41, 42]. Previous study[43] has found that preeclampsia is significantly related to homocysteinemia and insulin resistance. It is speculated that the pathological mechanism may directly damage endothelial cells through mechanisms such as hypomethylation, oxidative stress, and endothelial cell dysfunction, resulting in abnormal vasomotor function, which may be associated with the development of HDCP[44–46].

For decades, measures have been taken to reduce the dosage and improve the formula for the adverse reactions caused by the use of contraceptives. However, recent studies[47, 48] have shown that the use of low-dose contraceptives can still increase blood pressure. It is currently believed that the increase in blood pressure is mainly related to estrogen, and its mechanism may be: estrogen has the possibility of water and sodium retention[49, 50]. Estrogen can increase liver angiotensinogen. It is known that insulin resistance is closely related to hypertension, and estrogen can increase insulin resistance, which may be related to increased blood pressure. health screening for contraceptive users through training and guiding grassroots service personnel to implement informed choice of contraceptives and family planning follow-up services to prevent cardiovascular diseases in women, to improve the safety and efficiency of contraceptives[51, 52]. Meanwhile, it’s necessary to carry out in-depth research on the safety of contraceptives to further clarify the relationship between contraceptives and hypertension.

Several limitations in this present study must be concerned. Firstly, we did not calculate the sample size, the sample size may be not large enough to use logistic regression analysis, and it may be underpowered to detect the potential differences, future studies with larger sample size are needed. Secondly, since our study is retrospective analysis, many laboratory results could not be included for data analysis since some of included patients missing the related data, there might be some differences in the laboratory results. Besides, the distribution of PCOS phenotypes remained unclear in this study. which needs further investigations in the future studies. Thirdly, oral contraceptive pills are among the first-line treatment for menstrual disturbances and clinical hyperandrogenism in PCOS. The doctors asked patients for history of contraceptive pills usage and recorded it in the medical record, the types and dosage of contraceptive pills usage in our study remained unclear, which might introduce biases, therefore the results should be treated with cautions.

## Conclusions

In summary, we have found that the incidence of HDCP in patients with PCOS was rather high, and for PCOS patients with age ≥ 27y, BMI ≥ 24, family history of hypertension, the history of adverse pregnancy, history of contraceptive pills use and family history of HDCP, early alert for risk of HDCP development should be concerned, and associated preventions and treatments should be adopted to reduce the occurrence of HDCP. Obstetricians should provide education and guidance for PCOS patients before pregnancy, inform them of the risks of overweight or obesity during pregnancy, and take appropriate pre-pregnancy prevention measures, try to control the pre-pregnancy weight within the ideal range, avoid the adverse pregnancy, reduce the contraceptive pills use, strengthen pregnancy monitoring and screening, and formulate prevention and management measures to reduce the development of HDCP, to improve the maternal and child prognosis[53, 54]. However, the interrelationship between PCOS and HDCP and the underlying mechanisms are still unclear, which needs further investigations in the future.

## Data Availability

All data generated or analyzed during this study are included in this published article.

## Abbreviations

PCOS: Polycystic ovary syndrome
HDCP: Hypertensive disorders complicating pregnancy
BMI: Body mass index
ROC: Receiver operating characteristics
